# An Incidental Finding of a Massive Loculated Pericardial Effusion in a Patient Presenting With Inferior ST-Elevation Myocardial Infarction

**DOI:** 10.7759/cureus.62491

**Published:** 2024-06-16

**Authors:** Mustafa B Ozbay, Navin Bhatt, Catherine Duazo, Sean A Kotkin, Rosy Thachil

**Affiliations:** 1 Internal Medicine, New York Medical College/ Metropolitan Hospital Center, New York, USA; 2 Internal Medicine, NewYork City Health+Hospitals/Elmhurst, Mount Sinai School of Medicine, Queens, USA; 3 Cardiology, NewYork City Health+Hospitals/Elmhurst, Mount Sinai School of Medicine, Queens, USA; 4 Cardiology, Mount Sinai Fuster Heart Hospital, Icahn School of Medicine at Mount Sinai, New York, USA

**Keywords:** malignancy, end stage renal disease (esrd), st-elevation myocardial infarction (stemi), renal cell carcinoma, massive pericardial effusion

## Abstract

Pericardial effusion, commonly associated with malignancies such as lung, breast, and esophageal cancers through local extension, or leukemia, lymphoma, and melanoma via metastatic dissemination, is rarely observed in renal cell carcinoma (RCC). This report presents a rare case of a large loculated pericardial effusion in a 68-year-old male, potentially linked to RCC, who concurrently presented with an inferior wall ST-elevation myocardial infarction (STEMI). The patient, with a history of hypertension, hyperlipidemia, end-stage renal disease, coronary artery disease, and former smoking, exhibited symptoms including chest pain, diaphoresis, and shortness of breath, but no fever, chills, or night sweats. Diagnostic imaging revealed a significant pericardial effusion and a renal mass consistent with RCC, along with potential pulmonary metastases. Despite the complexity and high-risk nature of his condition, exacerbated by recent STEMI and dual antiplatelet therapy, a multidisciplinary approach was employed. This case emphasizes the need for careful management and tailored treatment strategies in patients with multiple coexisting conditions, highlighting the critical role of comprehensive diagnostic evaluation and collaborative care in improving patient outcomes.

## Introduction

Pericardial effusion, the accumulation of fluid in the pericardial sac, can be induced by various malignancies, including lung, breast, and esophageal cancers through direct local extension, and by others such as leukemia, lymphoma, and melanoma through metastatic dissemination [1]. These occurrences often result in a circumferential or global distribution of the effusion [1]. However, the incidence of pericardial effusion in renal cell carcinoma (RCC) is notably rare [1]. In this report, we present a case involving an elderly man with a potential diagnosis of renal cell carcinoma, who exhibited a large loculated pericardial effusion and concurrently presented with an inferior wall ST-elevation myocardial infarction (STEMI).

The pericardium, which encases the heart, can be involved through direct tumor invasion, lymphatic spread, or hematogenous dissemination [2]. Pericardial effusion in malignancy is often associated with poor prognosis, particularly when it occurs as a result of metastatic spread [3]. This condition can present asymptomatically or with symptoms ranging from chest pain and dyspnea to signs of cardiac tamponade, depending on the size and rapidity of fluid accumulation [4].

The rarity of pericardial effusion in RCC makes this case particularly notable [2]. Furthermore, the presence of both a significant cardiac event (STEMI) and a substantial pericardial effusion adds complexity to the clinical management of the patient, highlighting the interplay between cardiac and oncologic conditions. It also illustrates the challenges in managing such patients, especially when interventions are complicated by coexisting conditions and the need for careful consideration of treatment risks and benefits.

## Case presentation

**Figure 1:**
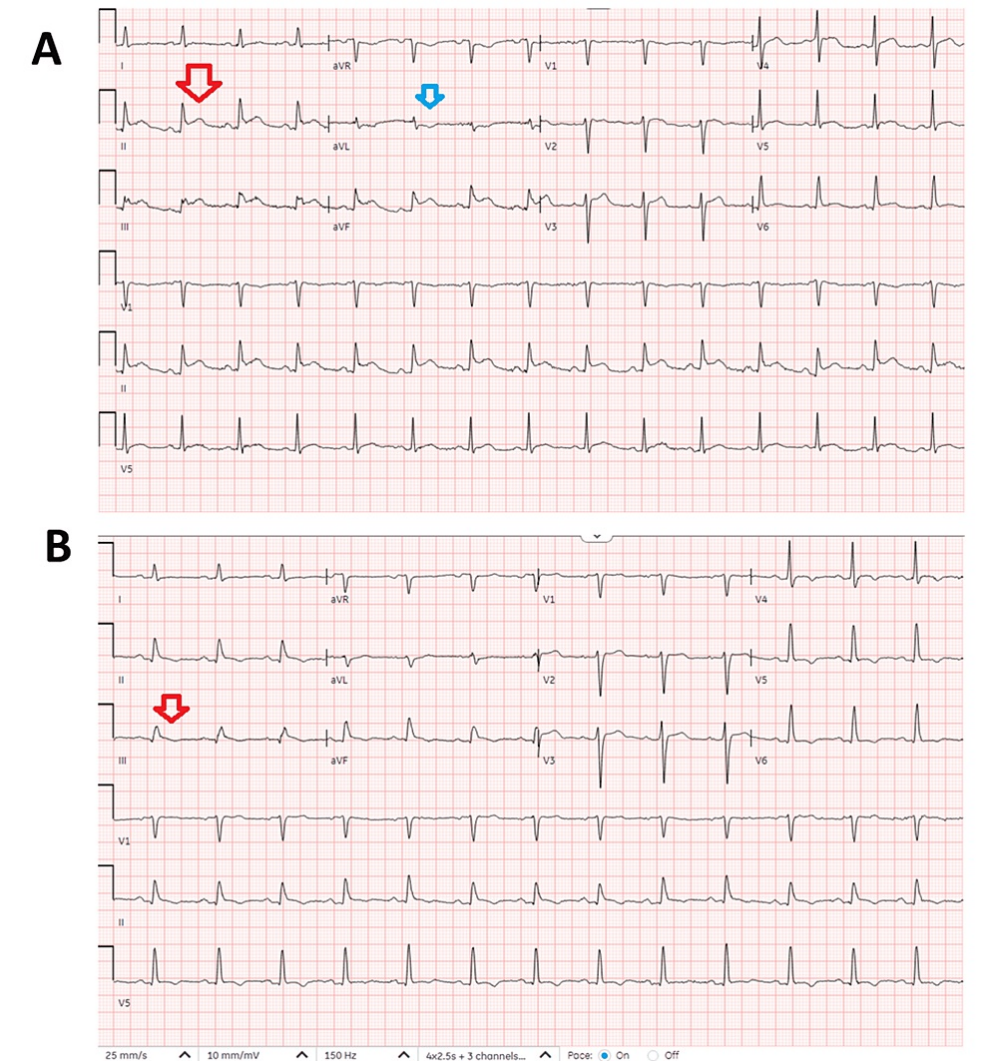
EKG consistent with STEMI. (A) EKG on presentation shows ST segment elevations (red arrow) in leads II, III, and aVF, and ST depression (blue arrow) in lead aVL. (B) EKG following the PCI procedure shows significant resolution of ST elevations in the inferior leads (red arrow) EKG: electrocardiogram, STEMI: ST Elevation Myocardial Infarction, PCI: percutaneous coronary intervention, aVR: augmented Vector Right, aVL: augmented Vector Left

**Figure 2:**
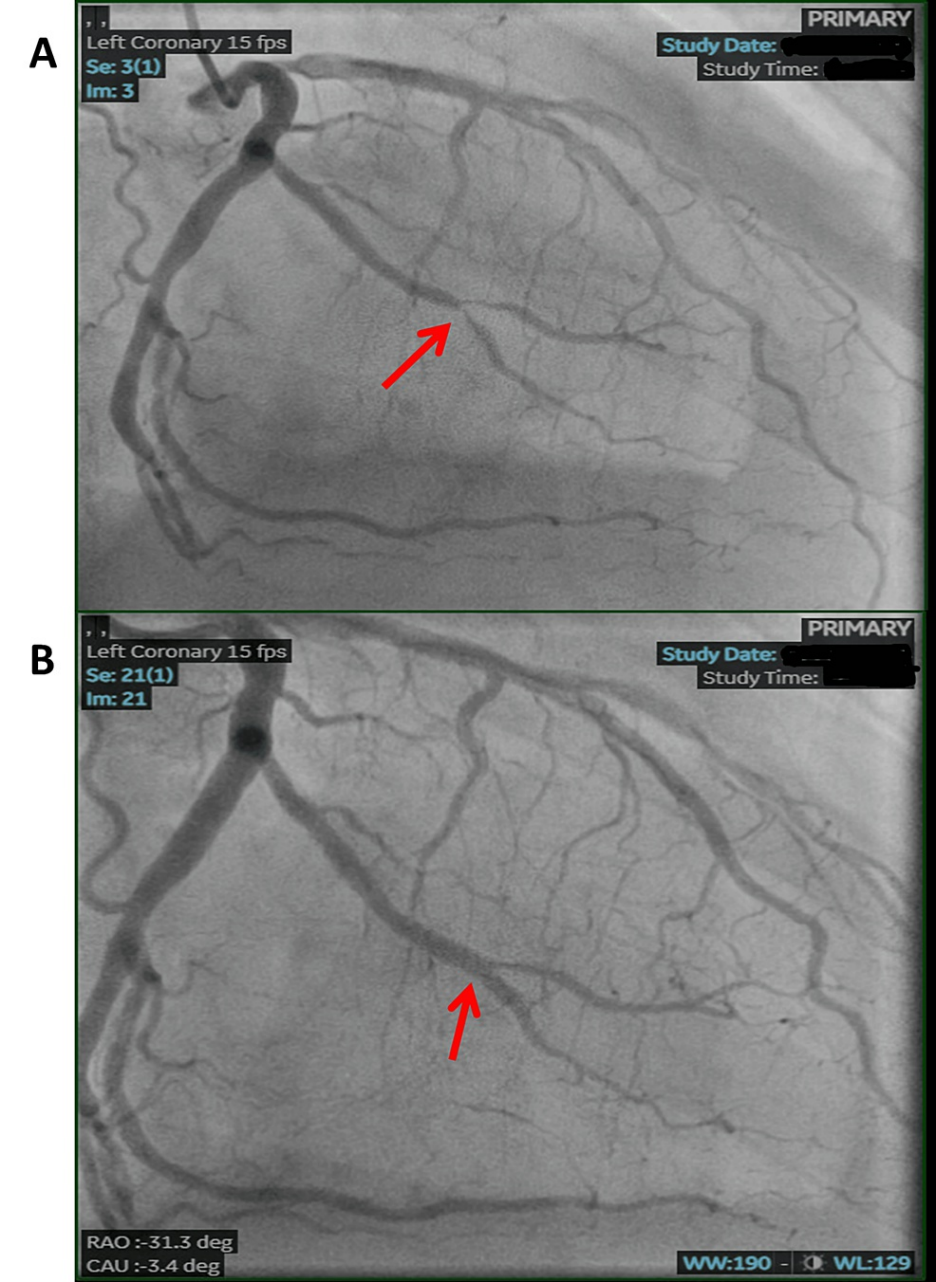
(A) LHC shows 90% stenosis of OM-2 (red arrow). (B) Post-PCI image of OM-2 (red arrow). LHC: left heart catheterization, OM: obtuse marginal, PCI: percutaneous coronary intervention

**Figure 3:**
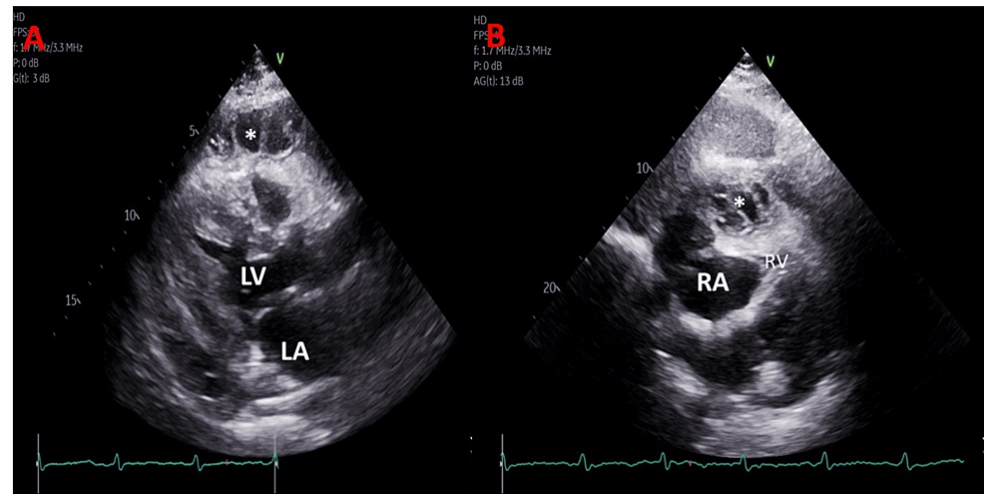
Transthoracic echocardiography shows pericardial effusion with multiple loculations (A) Parasternal long-axis view (B) Subcostal view. *pericardial effusion, LA: left atrium, LV: left ventricle, RA: right atrium, RV: right ventricle

A 68-year-old man with a past medical history of hypertension, hyperlipidemia, end-stage renal disease (ESRD) on peritoneal dialysis, coronary artery disease (CAD), and former smoker presented to the Emergency Department with worsening chest pain, diaphoresis, and shortness of breath. He stated that he hadn't experienced fever, chills, or night sweats, but had been consistently losing weight over the past year, approximately 15 pounds. The electrocardiogram (ECG) (Figure 1A) showed an inferior wall STEMI. The patient was taken to the catheterization laboratory for left heart catheterization (LHC). The right radial artery was accessed for primary percutaneous coronary intervention (PCI). LHC showed non-dominant right coronary artery (RCA) with proximal 85% stenosis, left anterior descending coronary artery with distal 50% stenosis, and first diagonal branch 50% stenosis and 90% stenosis of obtuse marginalis (OM)-2 (Figure 2A). The patient underwent successful placement of a drug-eluting stent (DES) (Figure 2B) in the OM-2, with significant resolution of ST-segment elevation (Figure 1B). The patient was transferred to the cardiac intensive care unit (CICU) after LHC. Transthoracic echocardiogram (TTE) showed basal and mid inferior left ventricular (LV) wall hypokinesis with an LV ejection fraction (EF) of 50%, large pericardial effusion with multiple loculations (Figure 3), abnormal respiratory ventricular motion (septal bounce), and an effusive-constrictive appearance. Additionally, there was no inferior vena cava plethora, early diastolic collapse of the right ventricle, or late diastolic collapse of the right atrium. There was no hepatic venous expiratory diastolic hepatic flow reversal or annulus reversus on TTE. Additionally, he had a TTE one month prior to presenting with inferior STEMI, which did not reveal any pericardial effusion.

**Figure 4:**
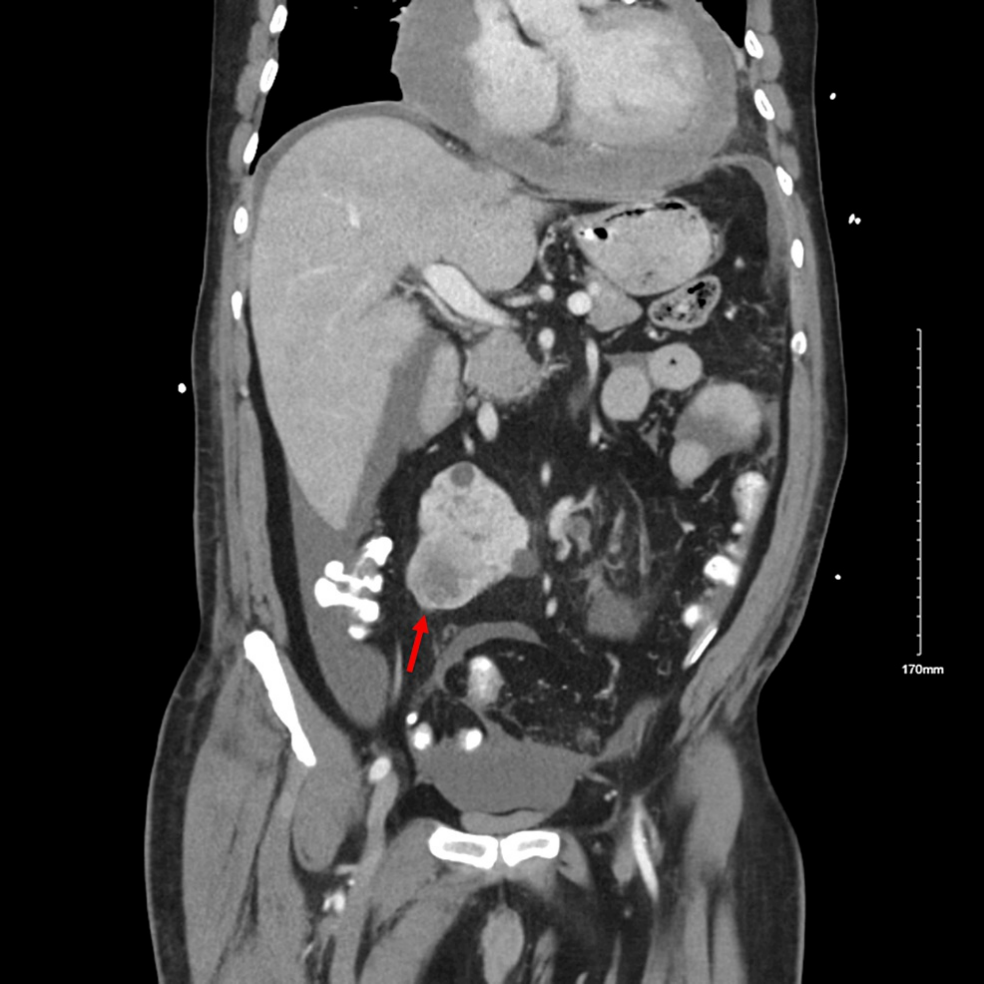
Sagittal CT abdomen/pelvis shows a 4 cm rounded heterogenous mass with some internal necrosis in the right kidney (red arrow). CT: computed tomography

**Figure 5:**
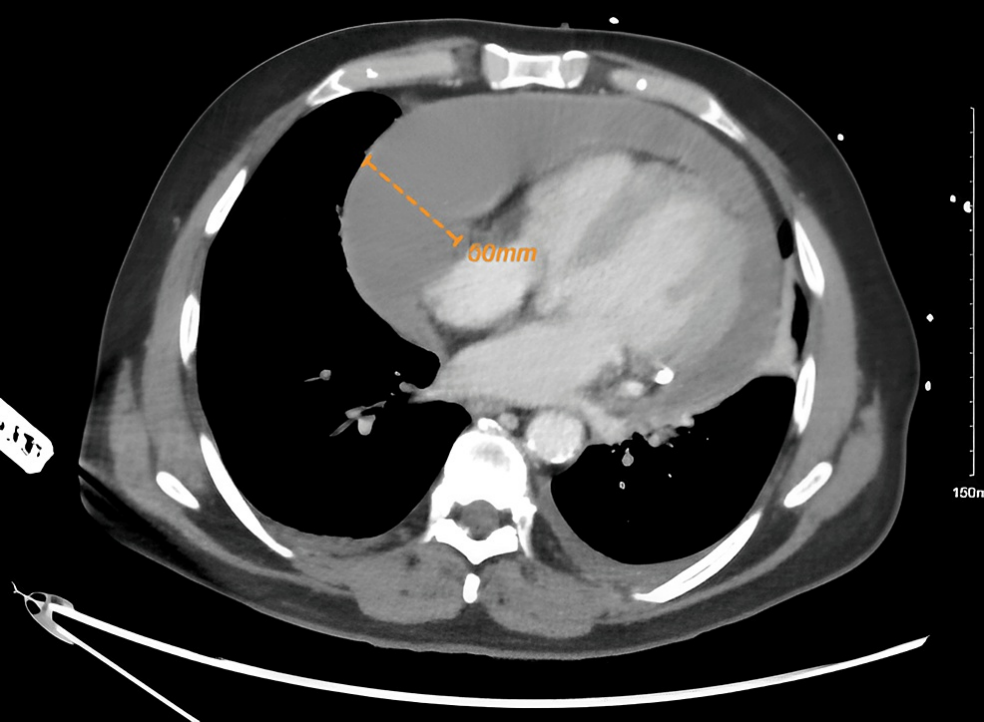
CT chest shows a large circumferential pericardial effusion measured up to 5 cm. CT: computed tomography

He had a Computed Tomography abdomen pelvis two months prior to presentation, just as a screening work-up for possible kidney transplantation, which showed a 3.6 cm heterogeneously enhancing mass in the right kidney consistent with renal cell carcinoma. He had been planned for nephrectomy. But in the interim time, he presented to our hospital for an inferior STEMI. We repeated the CT abdomen pelvis (Figure 4), which revealed a 4 cm right renal mass concerning for malignancy. CT chest was also done for possible metastasis, which showed scattered pulmonary nodules measuring up to 1.8 cm, likely metastatic in nature, and a large pericardial effusion (Figure 5) measuring up to 5 cm. Labs are demonstrated in Table 1. Tuberculosis was ruled out by three repeated acid-fast bacillus (AFB) sputum cultures and three Mycobacterium tuberculosis polymerase chain reaction (PCR) tests. The right heart catheterization (RHC) did not show elevated right-sided pressures. Pericardiocentesis and nephrectomy were deferred because of high risk due to the recent inferior STEMI and his being on dual antiplatelet therapy (DAPT). Lung biopsy for definitive diagnosis was also not performed due to the high risk of bleeding. The patient was discharged to follow up with Oncology as an outpatient and plan for tissue biopsy later to reduce the risk of bleeding and procedural complications following a recent STEMI.

**Table 1:** Selected labs on presentation and before the discharge BUN: blood urea nitrogen, WBC: white blood cell

	Day 0	Pre-discharge	Normal Range
Hemoglobin (g/dL)	9.0	9.7	13-17
WBC (/nL)	19.63	13.61	4.5-11
Platelets (/nL)	120	148	150-450
Na+ (mEq/L)	134	134	135-145
K+ (mEq/L)	4.5	4.0	3.6-5.2
CO2 (mEq/L)	22	23	23-29
BUN (mg/dL)	78	64	6-24
Creatinine (mg/dL)	10.60	8.66	0.7-1.3
Troponin (ng/L)	56	101	<14

## Discussion

Here, we present a case of incidentally found complex large pericardial effusion in a patient presented with inferior STEMI. Pericardial effusion can be categorized by its onset (acute or subacute vs. chronic if persisting for more than three months), distribution (circumferential, localized, or loculated), hemodynamic consequences (none, cardiac tamponade, effusive-constrictive), composition (exudate, transudate, blood), and notably, size [1]. Pericardial effusion is categorized as mild (<10mm), moderate (10-20mm), or large (>20mm) using semi-quantitative echocardiographic evaluation [3]. Our patient had a large circumferential pericardial effusion with multiple loculations and an effusive constrictive appearance.

Despite echocardiography being the main diagnostic method for pericardial diseases due to its wide availability, portability, and cost-effectiveness, CT and Cardiac Magnetic Resonance offer a broader field of view. This enables the identification of loculated pericardial effusion as well as thickening and masses within the pericardium [4]. CT chest showed a larger pericardial effusion than measured in TTE for our patient. However, TTE is superior to CT in assessing the hemodynamic implications of pericardial effusion, such as respiratory variations and septal bounce [1]. Septal bounce is defined as inspiratory ventricular septal motion toward the left ventricle [1].

Large pericardial effusions with frond-like projections, as observed in our case, indicate an exudative fluid but do not exclusively point to tuberculosis as the etiology. We sent three consecutive AFB sputum cultures, all of which were negative. If pericardial fluid cannot be obtained, a diagnostic score of 6 or higher, determined by the following criteria, strongly indicates tuberculous pericarditis: fever (1 point), night sweats (1 point), weight loss (2 points), globulin level exceeding 40 g/L (3 points), and peripheral leukocyte count less than 10x10^9/L (3 points) [1]. Our patient scored only 2 points, attributed to weight loss, which could potentially be indicative of malignancy as well. Therefore, we leaned more towards malignancy as the likely etiology of the pericardial effusion.

The prognosis of pericardial effusion depends primarily on its underlying etiology [3]. The size of the effusion affects the prognosis, as moderate to large effusions are frequently associated with specific causes like bacterial and neoplastic conditions [3,5]. The prevalence of malignancy for moderate to large pericardial effusion ranges from 4% to 40% depending on the studies referenced [1].

The recommended course of action for a large suspected malignancy-related pericardial effusion without tamponade consists of three treatments [1]. Systemic antineoplastic treatment serves as the foundational therapy [6]. Pericardiocentesis is performed to alleviate symptoms and establish a diagnosis. Injecting cytostatic or sclerosing agents into the pericardial space is done to prevent future recurrences [7-9]. The intrapericardial treatment should be customized based on the specific type of tumor [1]. Tetracyclines, when used as sclerosing agents, effectively control malignant pericardial effusion in around 85% of cases. However, they are associated with frequent adverse effects and complications, such as fever (19%), chest pain (20%), and atrial arrhythmias (10%) [6,10]. Pericardiotomy or pericardial window is recommended when pericardiocentesis is not feasible [11]. The patient presenting with an inferior STEMI and the need for DAPT complicated the workup. Thus, neither diagnostic studies such as tissue biopsy nor treatment modalities could be pursued immediately in our case due to the heightened risks associated with the recent STEMI and ongoing DAPT. Our patient did not have pericardiotomy since he is at very high risk for the surgery due to recent inferior STEMI and being on DAPT. Also, surgical pericardiotomy does not offer better clinical outcomes than pericardiocentesis and is associated with a higher occurrence of complications in malignant pericardial effusions [11]. In addition, clinical management often focuses on palliative care, aiming to relieve symptoms rather than treat the underlying condition in late-stage advanced malignancies, considering prognosis and patient quality of life [12].

In developed countries, the majority of effusive-constrictive pericarditis cases are idiopathic [13]. Tuberculosis is the predominant cause in developing nations [14]. The defining characteristic of effusive-constrictive pericarditis is the sustained elevation of right atrial pressure, even following the normalization of intrapericardial pressure through pericardial fluid drainage. RHC did not show elevated right-sided pressure, but the TTE study showed an effusive-constrictive appearance with septal bounce. A tissue biopsy is planned to be performed later to reduce the risk of bleeding and procedural complications following a recent inferior STEMI.

## Conclusions

Large pericardial effusion and myocardial infarction (MI) are rare as the initial presentation of a malignancy. Lung cancer is among the most common sites from which cardiac metastases arise. The majority of cases of neoplastic pericardial disease are not detected or diagnosed antemortem due to the usual lack of clinical symptoms. Cardiac metastases most commonly occur between ages 50 and 70 years, notably via lymphatic and hematogenous dissemination. This case underscores the importance of considering malignancy as a potential etiology in cases of acute pericardial disease. The presence of other concurrent risk factors may pose a diagnostic and treatment dilemma, potentially amplifying the risks of complications over the benefits. Therefore, adopting a multidisciplinary approach and devising a personalized treatment plan is crucial for enhancing patient outcomes.
